# Fabrication of biochar derived from different types of feedstocks as an efficient adsorbent for soil heavy metal removal

**DOI:** 10.1038/s41598-023-27638-9

**Published:** 2023-02-03

**Authors:** Marina Burachevskaya, Tatiana Minkina, Tatiana Bauer, Ilya Lobzenko, Alexey Fedorenko, Mahmoud Mazarji, Svetlana Sushkova, Saglara Mandzhieva, Alexander Nazarenko, Vera Butova, Ming Hung Wong, Vishnu D. Rajput

**Affiliations:** 1 Southern Federal University, Rostov-on-Don, Russia; 2 The Southern Scientific Centre, Russian Academy of Sciences, Rostov-on-Don, Russia; 3 Consortium On Health, Environment, Education, and Research (CHEER), and Department of Science and Environmental Studies, The Education University of Hong Kong, Tai Po, Hong Kong China

**Keywords:** Ecology, Environmental sciences

## Abstract

For effective soil remediation, it is vital to apply environmentally friendly and cost-effective technologies following the notion of green sustainable development. In the context of recycling waste and preserving nutrients in the soil, biochar production and utilization have become widespread. There is an urgent need to develop high-efficiency biochar-based sorbents for pollution removal from soil. This research examined the efficacy of soil remediation using biochar made from three distinct sources: wood, and agricultural residues (sunflower and rice husks). The generated biochars were characterized by SEM/SCEM, XRF, XRD, FTIR, BET Specific Surface Area, and elemental compositions. The presence of hydroxyl and phenolic functional groups and esters in wood, sunflower and rice husk biochar were noted. The total volume of pores was in the following descending order: rice husk > wood > sunflower husk. However, wood biochar had more thermally stable, heterogeneous, irregular-shaped pores than other samples. Adsorption of soil-heavy metals into biochars differed depending on the type of adsorbent, according to data derived from distribution coefficients, sorption degree, Freundlich, and Langmuir adsorption models. The input of biochars to Calcaric Fluvic Arenosol increased its adsorption ability under contamination by Cu(II), Zn(II), and Pb(II) in the following order: wood > rice husk > sunflower husk. The addition of sunflower husk, wood, and rice husk biochar to the soil led to an increase in the removal efficiency of metals in all cases (more than 77%). The increase in the percentage adsorption of Cu and Pb was 9–19%, of Zn was 11–21%. The present results indicated that all biochars functioned well as an absorbent for removing heavy metals from soils. The tailor-made surface chemistry properties and the high sorption efficiency of the biochar from sunflower and rice husks could potentially be used for soil remediation.

## Introduction

The soil provides 90% of human food, livestock feed, fiber, and fuel. Soil supplies raw materials and groundwater essential for life. Additionally, it serves as a habitat for billions of living species, including people. As a result, soils are vital resources in several of the 17 United Nations Sustainable Development Goals, including food security, clean water, improved sanitation, and excellent health and well-being^1^. The exponential rise in human population and significant growth in industrialization have resulted in the contamination of every ecosystem on Earth, especially soil. The soil contamination caused by heavy metals (HM) is a severe problem that reduces crop yields and negatively affects human and animal health^2^. The properties of HM make them non-biodegradable^3^. Therefore, HM remains intact in soil for a long time, necessitating discovering remediation methods to address the ever-growing soil contamination with HM.

Lately, environmentally sustainable and cost-effective remediation strategies for reducing HM's mobility and bioavailability in soils have been established^2,4,5^. For example, various organic and inorganic sorbents have been used to immobilize HM, transforming them into inaccessible fractions^6,7^. In this context, biochar is a highly effective and environmentally friendly sorbent with great potential for improving soil characteristics and recovering polluted soils^2,2,8^. Using biochar has favorable long-term outcomings on soils' such as improving pH, cation exchange capacity, and content of nutrients^9,10^.

Many studies have demonstrated the effectiveness of using carbonaceous sorbents to clean up HM-contaminated areas in agricultural land, mining, and urban environments have been shown in many studies^11–13^. Some researchers note the effectiveness of biochars made from various raw materials in the sorption and transformation of Zn in the soil^14^. Few studies revealed that HM adsorbed on biochar can be desorbed^15,16^. Biochar properties vary depending on feedstock and pyrolysis conditions. Optimizing production methods is critical for producing biochar that can be utilized to remediate contaminated soil efficiently.

Biochar can be fabricated from a variety of sources. The unequal efficiency and purity of biochar obtained from urban, industrial, food products, and agricultural waste are of great research interest and require further study. Despite the variety of raw materials for biochar manufacturing, industrial waste, effluents, and waste products from farmed animals have drawbacks, such as complex and economically costly pyrolysis and doubts about their purity and environmental safety. Converting crop residues into biochar is one of the sustainable answers to the realization of soil remediation, boosting agricultural output, improving soil fertility, and sequestering carbon. Processing agricultural waste into biochars was recommended as a cost-effective and environmentally friendly treatment and resource utilization method^17,18^. Reusing leftover biomass instead of disposing of it has the added benefit of reducing its environmental impact^19^. Given these limitations, plant biomass is the most valuable raw material, divided into woody and non-woody^20,21^. Woody biomass is known for its low moisture and ash contents, high bulk density, and low porosity, while non-wood biomass mainly entails crops and their residues. A BET Specific Surface Area characterizes the most common biochar obtained from wooden residues to the peculiarities of the wood structure^20,21^.

Biochar manufacturing from crop residues (non-woody) by pyrolysis is one of the most promising processing methods, as confirmed by its high BET specific Surface Area and large porosity, slightly inferior to wood analogues^22^. One of these wastes is rice husks and sunflower husks. In 2019/2020, the harvest of sunflower grain in the world amounted to 55.9 million tonnes, and the raw rice harvest reached 501.1 million^23^. The share of rice and sunflower husks in the grain yield averages 20% by weight^24^. Although rice husks and sunflower husks are widely used in various fields, the accumulation of rice husks is estimated to be 17 thousand tonnes per year, and sunflower husk production reaches 361 thousand tonnes per year in the Rostov region (southern part of Russia)^24^.

Despite the widespread use of sunflower husks as additives to animal feeds and building materials and rice husks for silica production, crop waste accumulates in significant volumes, posing problems for their environmentally safe disposal. An example of such waste disposal generation is sunflower oil production by the hot-pressing method, with the husk waste forming up to 11–16% by weight of the raw material. The amount of oil husks that may be produced every day varies widely depending on the capacity of the oil extraction or pressing facility. Most of these husks are disposed of in landfills though some are used as animal feed.

Traditionally, rice husks are stored in landfills or burned at rice production sites. However, rice husk may also be used as raw material for making and producing new compounds with a high added value, such as carbon-containing materials. Husk has low bulk weight, flammability, and smoldering propensity contributing to an unpleasant odor and significantly negatively impacting the environment. In light of this, there is a need to develop and apply new technologies for these plant-based materials rather than open burning or landfill disposal, which eventually damage the environment due to the release of highly toxic combustion products. Therefore, the crop residues should be recycled and reused to achieve environmentally appropriate agricultural management. Sequestering carbon in soil by biomass conversion to biochar has been considered one of the best methods of mitigating climate change^25,26^. In this regard, biochar has the potential to build a zero-waste policy for sustainable farming, thus, decreasing the associated pollution loading to the environment. To suit management requirements explicitly for soil, the biochar's physicochemical properties, structural features, and pyrolysis circumstances must be entirely determined and optimized. The raw materials and cases of pyrolysis determine functional groups that facilitate the surface complexation of HM cations. The surface binding of polar pollutants primarily depends on groups such as hydroxyl, aldehyde, and ketone^27,28^.

In addition to the presence and composition of functional groups, BET specific Surface Area and porosity, mineral content, and cation exchange capacity will all impact biochar's sorption properties and affinity towards different contaminations^29^. Recent research has also shown that biochar adsorption capabilities for HM vary greatly depending on biochar characteristics and the nature of the target metal^30,31^. In this regard, assessing the adsorption of pollutants by soil in the presence of biochars that differ in the porous structure, composition, and properties is essential. Thus, biochars obtained from various biomass feedstocks have different efficiencies and mechanisms in immobilizing HMs and other pollutants. This paper considers the surface area parameters of biochars obtained from various raw materials.

Most research has looked at the one target metal ion as a whole; therefore, it is essential to perform the adsorption of various HM. This study aimed to compare the effect of types of feedstocks on the sorption properties of biochars obtained from agricultural wastes and wood used for the removal of HM-contaminated soil.

## Results

### Elemental analysis

It was found that the biochar samples had similar indicators in the elemental percentage, as shown in Table 1. As given in Table 1, the ranges of C, N, H, and O were 70.4–77.3%, 2.1–2.4%, 3.0–4.8%, and 7.3–11.8%, respectively.

**Table 1:** Average elemental composition of the studied biochars.

Biochar type	Element and ash content, %					Atomic ratio			
	C	N	H	O	Ash	H/C	O/C	C/N	(N + O)/C
Wood	77.3	2.4	4.8	7.3	8.2	0.75	0.07	37.9	0.10
Sunflower husk	73.5	2.2	3.4	10.0	10.9	0.56	0.10	38.3	0.13
Rice husk	70.4	2.1	3.0	11.8	12.7	0.51	0.13	39.1	0.15

### XRF

The XRF results of different biochar samples are shown in Supplementary materials (Table S1). Determining the elemental composition of biochar in the samples did not exceed the maximum permissible concentrations of trace HM (even for raw food materials and food products^32^. The proportion of SiO_2_ in the biochar from rice husks was higher (47.52%) than that in the other studied samples. In addition, high contents of phosphorus (20.96% P_2_O_5_) and potassium (24.33% K_2_O) were observed in sunflower husk biochar, as well as a high calcium content in wood biochar (26.30% CaO).

### XRD

**Figure 1:**
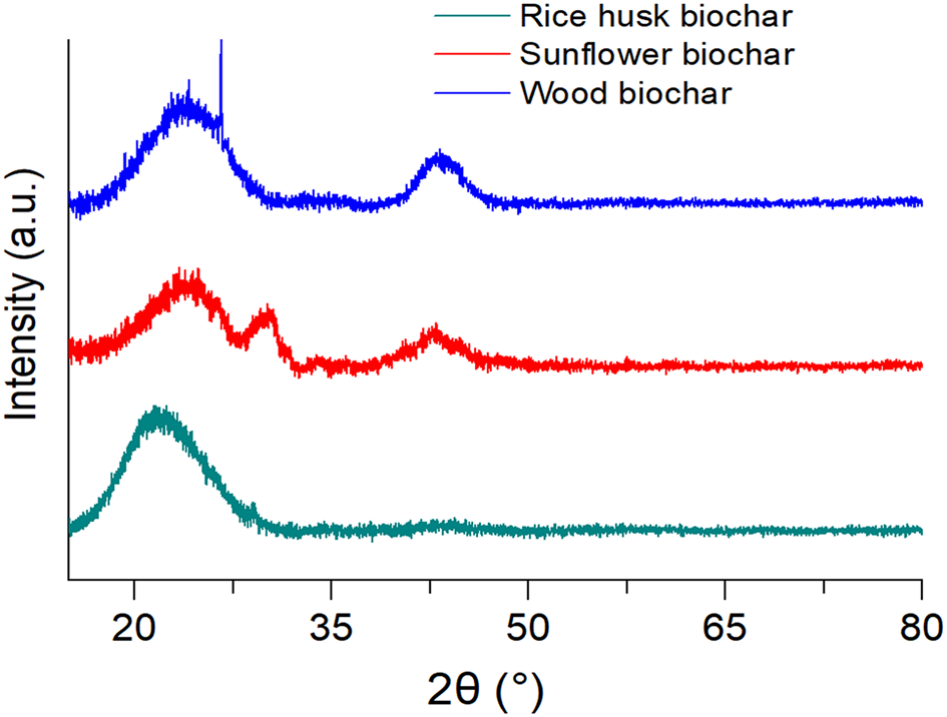
XRD spectra of biochars from various raw materials.

The crystal structures of various biochar samples were investigated. As shown in Fig. 1, rice husk biochar contained only a wide projection centered at 20°. Moreover, in sunflower husk biochar, the peak was detected at 2θ = 24.40°, as well as two other peaks at 2θ = 30.57° and 42.67°. The rice husk biochar showed a similar pattern to biochar from sunflower husks, except for the disappearance of the peak at 42.67°. In addition, the spectrum of wood biochar showed distinct peaks from other samples at 2θ = 22°and 2θ = 41°.

### FTIR

**Figure 2:**
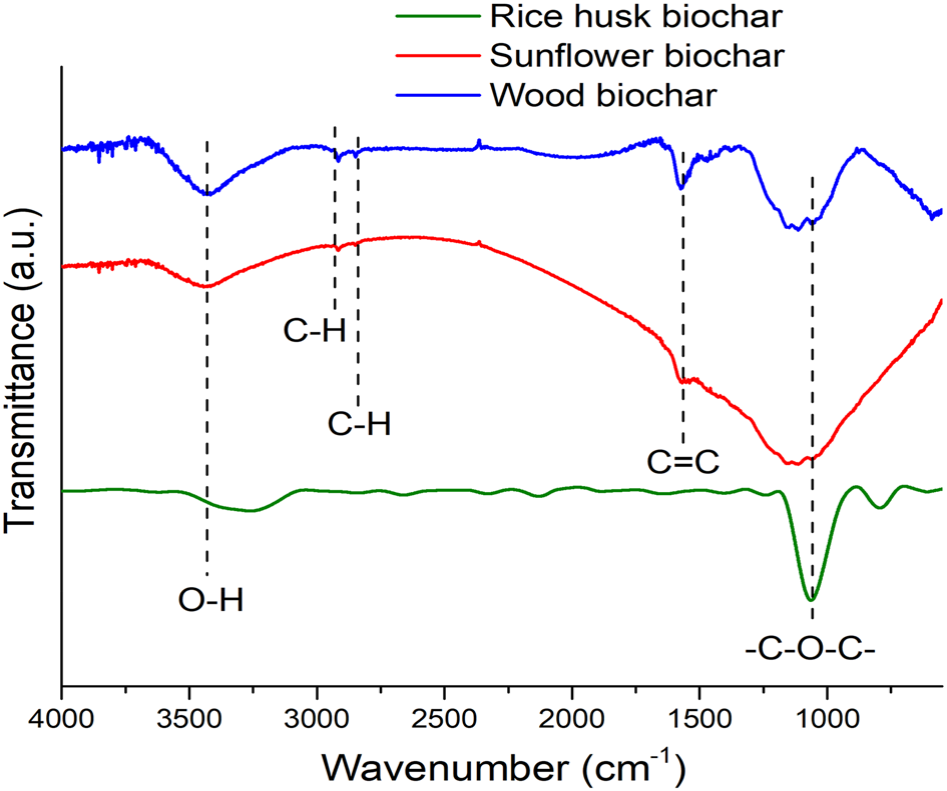
FTIR spectral characteristics of biochar from various raw materials.

The FTIR spectra of wood biochar in Fig. 2 showed a strong peak at around 3100–3600 cm^−1^, corresponding to OH stretching vibrations from the hydroxyl functional groups or adsorbed water. The narrow peaks at 2918 and 2846 cm^−1^ are probably associated with the asymmetric and symmetric stretching vibrations of the aliphatic C-H groups of cellulose, respectively. The bands at approximately 1570 and 1476 cm^–1^ are attributable to the stretching vibrations of C=C and bending vibrations of C–H, respectively. The strong and broad peak at 900–1250 cm^-1^ was attributed to the symmetric valence groups –C–O–C– or phenolic functional groups and ethers. The FTIR spectra of biochar obtained from sunflower husks (Fig. 2) showed a strong peak at 3100–3600 cm^–1^ (stretching vibrations of the OH and adsorbed water) and at 900–1250 cm^–1^ attributed to C–O–C bond. The FTIR spectra of the rice husk biochar were similar to the sunflower husk sample, where the peaks at 3485 cm^-1^ and 1071 cm^-1^ corresponded to OH and C–O–C, respectively.

### SEM

**Figure 3:**
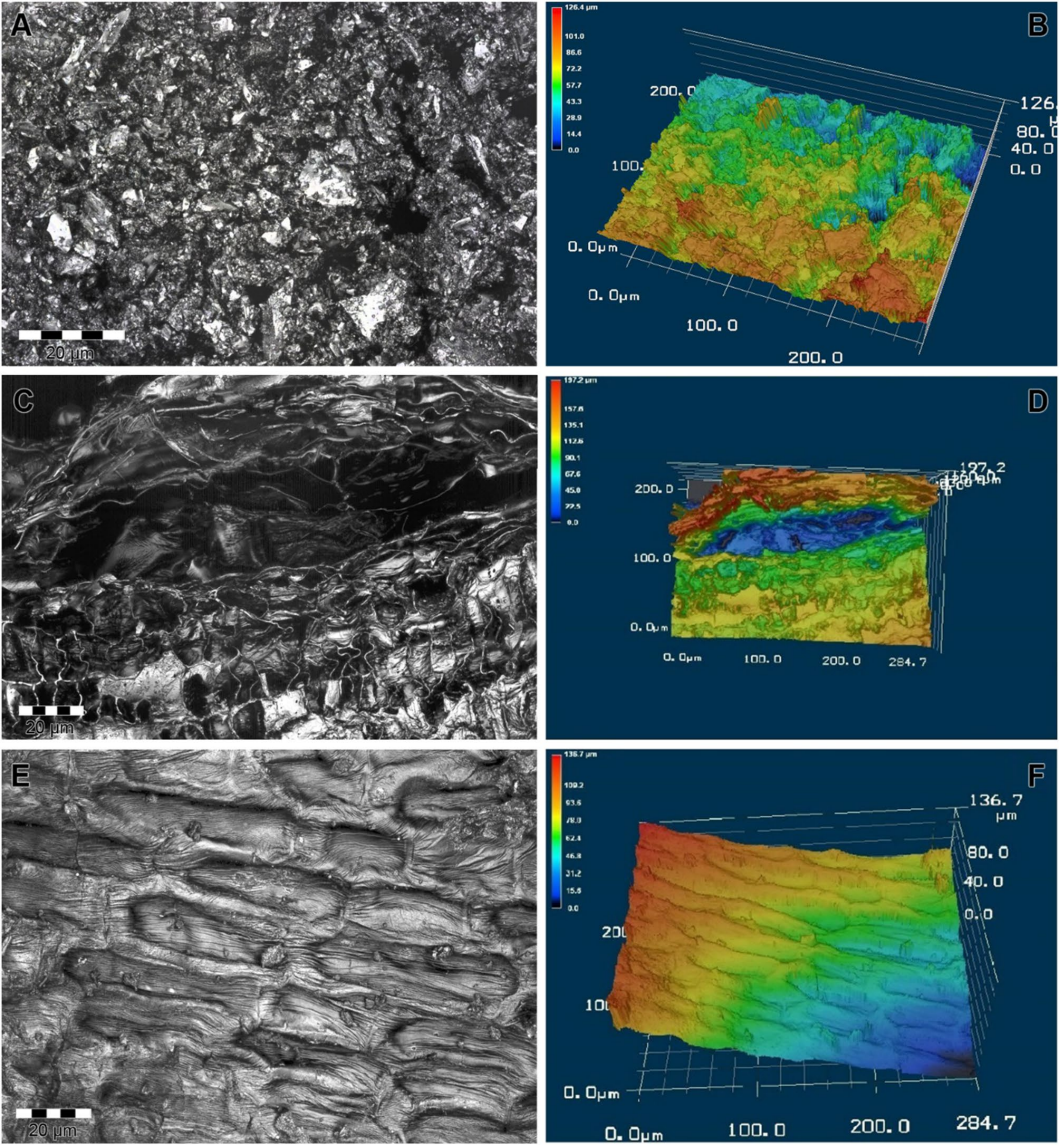
SEM images of different biochars: (**A**) wood; (**B**) 3D model of the wood biochar surface; (**C**) sunflower husks; (**D**) 3D model of sunflower husk- biochar surface; (**E**) rice husks; (**F**) 3D model of the surface of rice husks.

The SEM measurements, supplemented by 3D modelling, showed that the sorbents have a biogenic structure inherited from the raw starting materials (Fig. 3A,C,E). The microscopy images showed that the cellular morphologies of wood maintained by biochar (Fig. 3A,B) was heterogeneous and can be grouped into fibrous, prismatic, and spherical structures. Moreover, the pores were irregular in shape (Fig. 3A). The size of the individual particles ranging from 1 to 10 µm. There are monolithic inclusions up to 50 µm in size, which are the remains of the vascular system of wood. The surface relief of the particles was not uniform. The structure of biochar from sunflower husks is anisotropic. In the sagittal plane, biochar particles from the husk have longitudinal slit-like engagement and bulges. In the cross-section, they represent a system of pores from 5 to 15 μm formed by the cell walls of the starting material (Fig. 3C,D).

The structure of the rice husk biochar (Fig. 3E,F) was similar to that of the sunflower husk biochar. It was also anisotropic along the longitudinal axis. The surfaces lying in the sagittal plane were smooth with a relatively shallow relief. The surfaces lying in the transverse plane are represented by large invaginations 10–50 µm in size, owing to the cellular structure of the original plant material.

### BET

Biochar manufactured from rice and sunflower husks had a slightly lower BET Specific Surface Area than from wood. The lowest pore volume (ΣV) was related to the biochar from sunflower husks (2.50 cm^3^/g), and the highest was found in rice husk biochar (2.88 cm^3^/g) (Table 2). The volume distribution between micro-, macro-, and mesopores for biochar from rice and sunflower husks was identical. The most significant volume was mesopores (1.59 cm^3^/g for biochar from rice husks and 1.65 cm^3^/g for sunflower husks). A proportion of the volume was significantly related to macropores (1.31 cm^3^/g) followed by mesopores (1.14 cm^3^/g) in the wood biochar sample. Moreover, the smallest volume of micropores was typical for biochar made of wood, where the volume of micropores was 0.28 cm^3^/g.

**Table 2:** Physical characteristics of the biochar samples.

Biochar type	Mean particle size (mm)	BET specific Surface Area (m^2^/g)	Pore volume, V (cm^3^/g)			
			ΣV	V_macro_ > 50 nm	V_meso_ 2–50 nm	V_micro_ ˂2 nm
Wood	0.5–4	612 ± 25	2.73	1.31	1.14	0.28
Sunflower husk	1–5	353 ± 11	2.50	0.35	1.65	0.50
Rice husk	1–3	198 ± 8	2.88	0.68	1.59	0.61

### TGA

**Figure 4:**
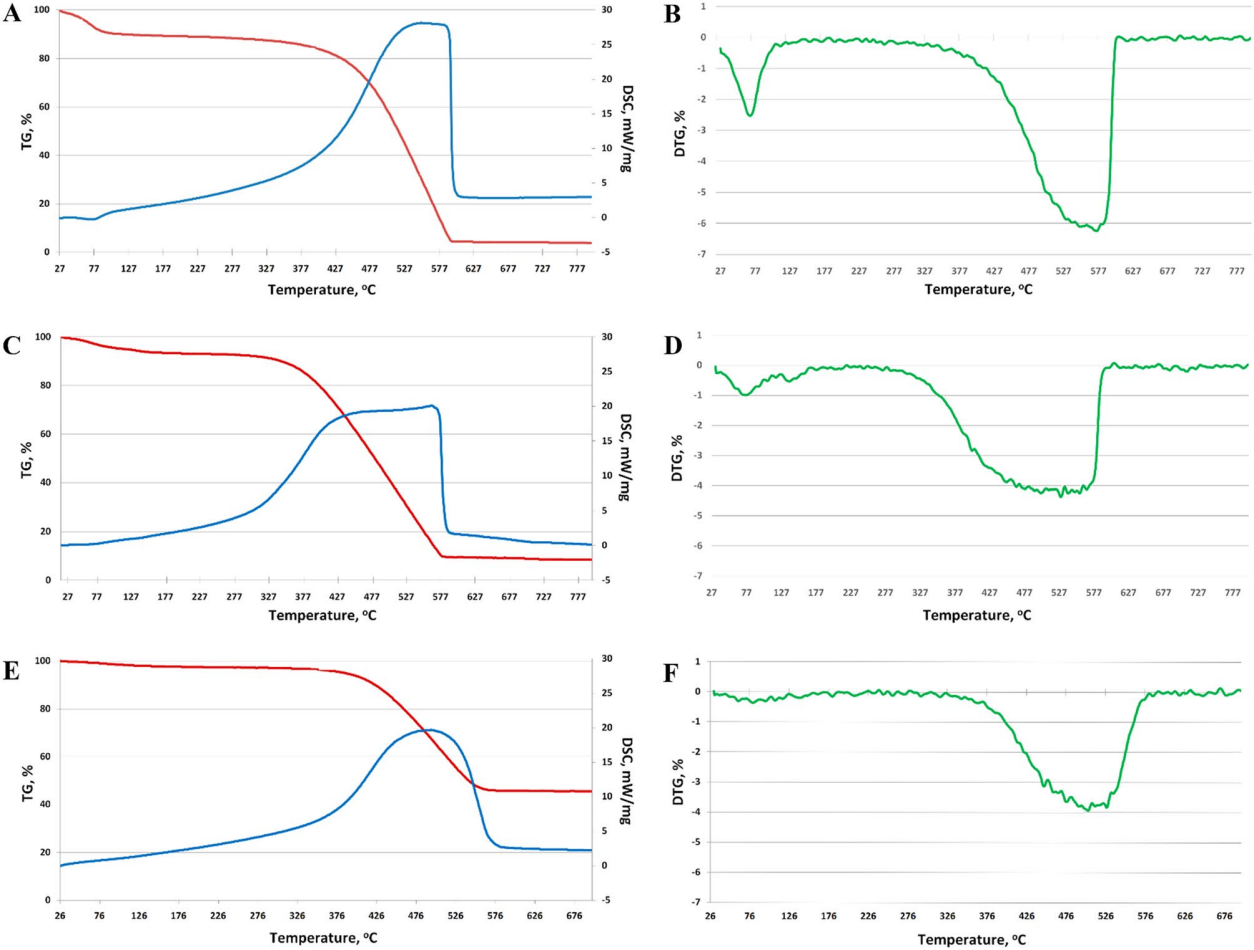
TGA (red line) and DSC (blue line) results from various biochar: (**A**) wood; (**C**) sunflower; and (**E**) rice husk; and DTG (green line) result from (**B**) wood; (**D**) sunflower husk; and (**F**) rice husks.

The results of STA wood biochar showed that heating the sample at 95–100 °C removed hygroscopic water, sorbed gases, and various volatile low-molecular-weight organic compounds from the sorbent surface (TGA curve). This is displayed on the DSC graph as an endothermic process (Fig. 4A) and the DTG curve (Fig. 4B) for a given temperature range. The weight loss of the sample at this stage was 10%. When biochar was heated to 250–300 °C, gradual combustion of residual fragments of lignin and cellulose began, which intensified at 390–400 °C owing to the amorphous carbon oxidation of the main component of biochar. This exothermic process was observed in the DSC curve (Fig. 4A). This was accompanied by a significant and most intense weight loss (Fig. 4A,B), which, upon reaching 600 °C, amounted to 84%. The total weight loss of the sample was 96%.

The results of TGA for biochar from sunflower husks showed that at temperatures of approximately 105–110 °C, the main mass losses were associated with the desorption of moisture from the sorbent surface (Fig. 4C). Also, some residual volatile organic compounds evaporated, such as, for example, naphthalene in the range < 150 °C. The weight loss of the sample at this stage was approximately 6%, slightly less than that of the wood biochar. The next stage of biochar decomposition was confined to the interval of 350–580 °C, associated with the process of carbon oxidation. This process was exothermic and related to releasing a large amount of energy, observed in the TGA curve (Fig. 4B).

The most extreme weight loss was recorded at this stage, accounting for 80% approximately (Fig. 4D). The total weight loss during the simultaneous thermal analysis of the biochar from the husks was 92%.

The sample from rice husks was also characterised by insignificant weight loss in the temperature range of 105–125 °C. This was associated with removing adsorbed water and some volatile compounds that were by-products of the pyrolysis. However, the weight loss in this temperature range was 2%, which indicated a lower hygroscopicity of this biochar and a lower content of volatile compounds. An insignificant weight loss was observed at temperatures of 230–250 °C, associated with the oxidation of cellulose residues (Fig. 4E,F). However, due to the high carbonisation of biochar, its weight loss at these temperatures did not exceed 3%. The final stage of thermal decomposition of the biochar from rice husks refers to the combustion of carbon, which begins at a temperature of 375 °C. This process was characterised by the most intense weight loss (Fig. 4E), which amounted to 50%. The total weight loss was 54% of the initial sample weight, the smallest of all studied samples.

### The use of biochar for the remediation of heavy metal-contaminated soil

**Figure 5:**
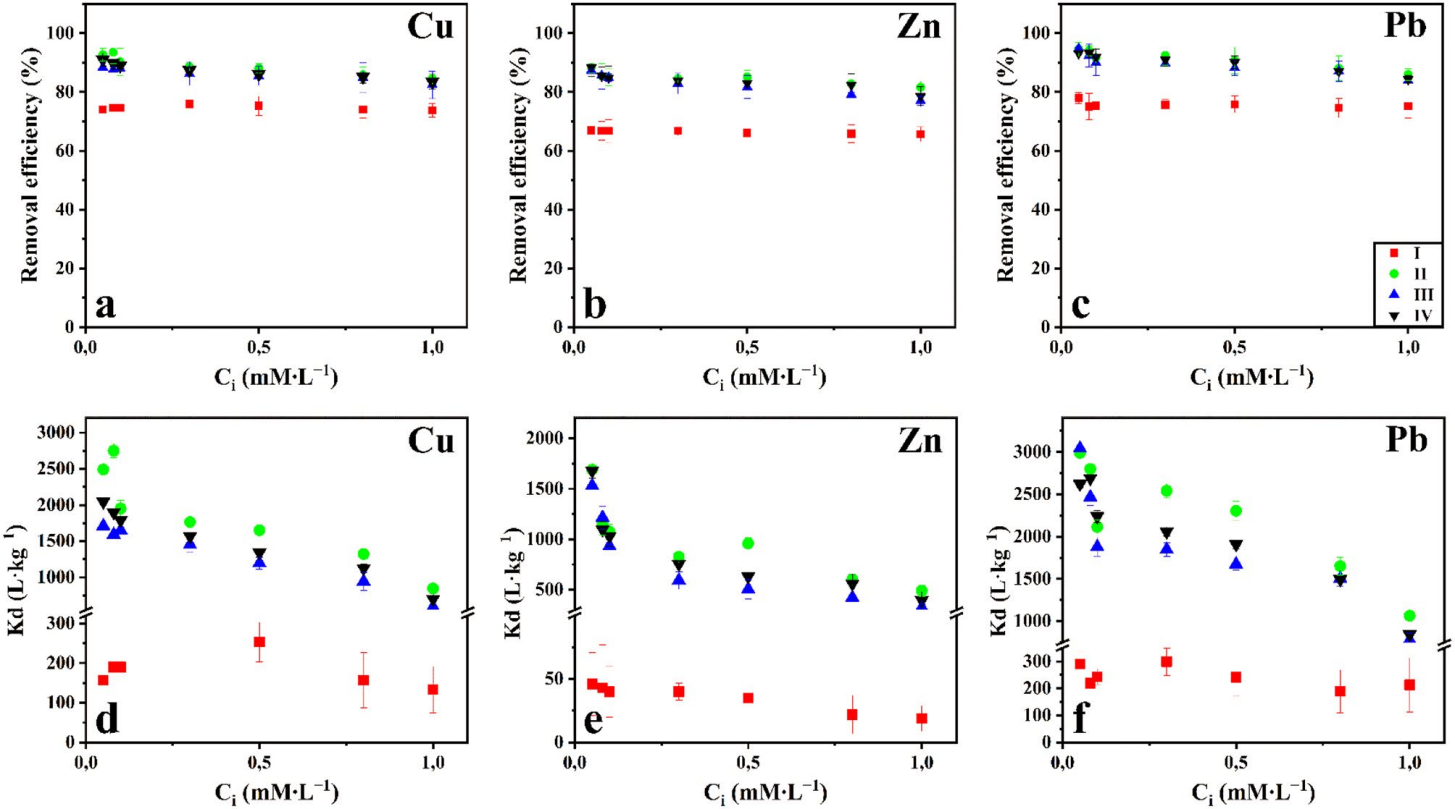
The equilibrium sorption coefficient (**a**–**c**) and removal efficiency (**d**–**f**) for the different variants of treatments (soil (I), soil + Wood biochar (II), soil + Sunflower husk biochar (III), soil + Rice husk biochar (IV)).

Figure 5 shows the equilibrium sorption coefficients (Kd) and removal for Cu(II), Zn(II), and Pb(II) ions. The results showed different adsorption capacities due to the differences in the chemical properties of the studied biochars.

In solutions with an initial metal content of up to 1.0 mM, the removal efficiency value of Cu(II) ions was 74–76%. The percentage removal of Pb(II) sorption decreased from 78 to 75%. In contrast, for Zn(II), it fell from 67 to 66% for the entire concentration range (Fig. 5). The Kd values obtained for Pb(II) were significantly higher than the established values for Cu(II) and, especially for Zn(II). All the treatments had an increase in initial metal concentrations and a decrease in Kd and removal efficiency.

The addition of sunflower husk, wood, and rice husk biochar to the soil led to an increase in the removal efficiency of metals, which was more than 77% in all cases (Fig. 5). Under all types of biochar, the increase in the percentage adsorption of Cu and Pb was 9–19%. In the case of Zn, the highest increase was observed in the range of 11–21%. The highest values of S were observed for Pb (II) (up to 95%). When all types of biochars were added to the soil, the Kd value increased by 4–15 times for Cu, 15–37 times for Zn, and 4–10 times for Pb magnification (Fig. 5).

**Figure 6:**
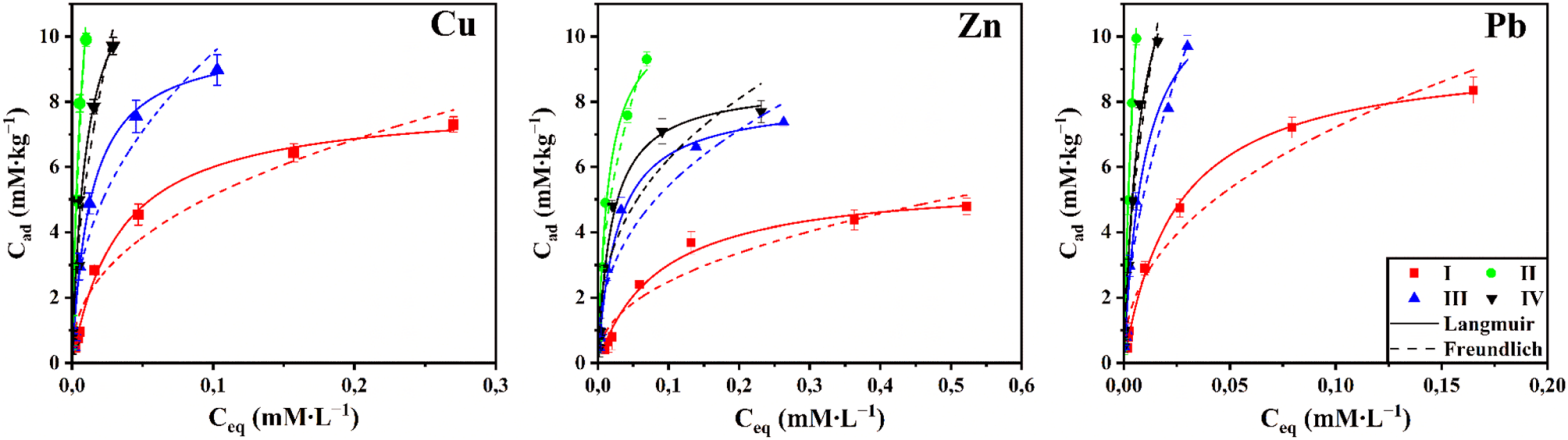
Adsorption isotherms of HM by soil in pure form (I) and soil with different biochars (soil + Wood biochar (II), soil + Sunflower husk biochar (III), soil + Rice husk biochar (IV)).

The isotherms data of HM (Fig. 6) were fitted better to the Langmuir model in all cases, with R2 values greater than 0.98 (Table 3). The highest C_m_ and K_L_ values were found for Pb(II) (Table 3). The C_m_ and K_L_ values of Calcaric Fluvic Arenosol for HM cation were seen in the following order: Pb(II) > Cu(II) > Zn(II).The values of the K_F_ coefficient for the soil change in the following sequence: Pb (19.67 L∙kg^−1^) > Cu (13.35 L∙kg^−1^) > Zn (6.82 L∙kg^−1^). In general, using both sorption models leads to the same conclusion: the sequence of adsorbate location depending on the value of C_m_, calculated according to the Langmuir equation, and K_F_, is similar (Table 3). At the same time, the values of K_F_ in all cases are higher than the calculated values of C_m_, especially for Cu(II) and Pb(II).

**Table 3:** Fitted parameters for adsorption isotherms of HM by soil in pure form and with different biochars in single-metal solutions.

Sample	Langmuir			Freundlich		
	C_m_ (mM∙L^–1^)	K_L_ (L∙mM^–1^)	R^2^	K_F_ (L∙kg^–1^)	n	R^2^
**Cu(II)**						
Soil	8.01	29.76	0.997	13.35	0.41	0.959
Soil + Wood biochar	17.69	135.17	0.983	236.67	0.68	0.963
Soil + Sunflower husk biochar	9.98	76.45	0.998	25.15	0.42	0.950
Soil + Rice husk biochar	13.15	97.64	0.976	73.48	0.56	0.948
**Zn(II)**						
Soil	5.60	11.57	0.988	6.82	0.44	0.918
Soil + Wood biochar	10.91	67.05	0.990	33.46	0.47	0.980
Soil + Sunflower husk biochar	8.10	36.61	0.991	13.55	0.40	0.923
Soil + Rice husk biochar	8.55	50.42	0.985	14.87	0.38	0.881
**Pb(II)**						
Soil	9.53	40.19	0.998	19.67	0.43	0.959
Soil + Wood biochar	20.74	158.29	0.994	447.13	0.73	0.986
Soil + Sunflower husk biochar	12.22	105.40	0.983	66.41	0.55	0.969
Soil + Rice husk biochar	15.04	126.64	0.989	111.76	0.57	0.969

The addition of biochars obtained from different feedstocks leads to an increase in the bond strength of HM with soil: the K_L_ value increases 2.8–4.5 times for Cu(II), 3.2–5.8 times for Zn(II), and 2.6–3.9 times for Pb(II) (Table 3). The adsorption capabilities of the biochars for Cu(II), Zn(II), and Pb(II) were wood > rice husk > sunflower in all of the experiments.

## Discussion

The theoretical explanation for soil's higher absorptive capacity concerning HM in biochar's presence lies in its chemical and physical properties, including functional groups, mineral contents, zeta potential, surface area, and pore volume^33^. The latter mainly depends on the features of the sorbent texture, such as the BET Specific Surface Area and pore structure. The raw materials used to define the listed features of the sorbent texture that affect its physico-chemical properties and sorption abilities. The texture characteristics of sorbents strongly depend on plant type, growing conditions, and biochar production parameters, which vary in extensive ranges. Rice husk biochar is characterized by a range of BET Specific Surface Area from 34 to 774m^2^/g^34–36^ for sunflower residues—from 7 to 434 m^2^/g^22,37,38^ for wood raw materials—from 6 to 557 m^2^/g^39–41^. Thus, the biochars used in this study are characterized by high texture indicators, suggesting the efficacy of raw materials' applied pyrolysis scheme.

Based on the atomic ratio of the elements obtained by the elemental analysis method, we can assume that the system of conjugated aromatic bonds is well developed in biochars. The degree of aromaticity indicates that aromatic compounds prevail in the studied sorbents, mainly in wood biochar. According to another study^42^ biochar with a high C: N ratio promotes the immobilisation of microbial nitrogen. This will lead to a decrease in the flow of greenhouse gases and an increase in organic carbon content in the soil.

According to XRF analysis, the high silicon content in the rice husks biochar was observed. This feature is due to the raw material Si content^43^. This also indicates a higher ash potential of biochar from rice husks. Rice production wastes differ in chemical composition from all other raw materials, primarily in their high content of amorphous silicon dioxide. These features are associated with the different accumulations of elements and various plant raw materials.

The XRD method established that the biochar made from rice husks showed a wide hump with a center at an angle of 20°, indicating the presence of SiO_2_^5,44,45^. In the biochar sample from sunflower husks, the protrusion at 2θ = 24.40° belongs to the quartz phase, and the other two protrusions, to the calcite phase^46–48^. The biochar from wood contained the same phases as in the previous sample, and the phase of crystalline cellulose was also detected.

TGA data showed that the rice husks biochar was the most carbonated of all samples studied, what is related to low lignin content of rice husks and also had the lowest number of functional groups. Biochar from wood contained significant amounts of cellulose and lignin, which were not degraded during pyrolysis. Sunflower-husks biochar occupied an intermediate position. Lignin is a complex phenolic polymer comprising three alcohol monomers (n-coumarol, coniferyl alcohol, and sinapyl alcohol). Its biomass content ranged from 15 to 30%^20^.

The lowest content of functional groups in rice husk biochar is also concentrated on high ash. As the charring intensity increases, ash works as a heat-resistant component, potentially protecting organic molecules from degradation while preventing aromatic structure production^49^. The FTIR spectroscopy showed that wood biochar has a more hydrophilic surface, while rice husk biochar has the most hydrophobic surface. Wood biochar contains more hydrophilic functional groups, which enhances its effect on soil moisture permeability^50^.

Microscopic data showed that biochars from various raw materials inherit the biogenic structure of the starting materials. The surface relief of the particles in the studied samples was inhomogeneous. However, depending on the raw material, the degree of surface heterogeneity of the biochar differed. Three-dimensional modelling of biochar samples showed (Fig. 3) that wood has the most developed surface, followed by biochar from sunflower husks, and the least developed micro relief of biochar from rice husks. These data are consistent with the BET Specific Surface Area measurements using the adsorption–desorption analysis of N_2_.

The results of determining the BET Specific Surface Area and porosity were consistent with the microscopy results. Wood biochar's highest BET Specific Surface Area was observed because its surface was the most heterogeneous. In addition, this sample was characterised by the most significant volume of macropores, which is consistent with the microscopic data. Rice husk biochar has less BET Specific Surface Area than others because its relief is the most ordered. According to these indicators, biochar from sunflower husks occupies an intermediate position^51^.

Compared with agricultural herb waste (sunflower and rice husks), wood source materials are rich in cellulose and easy to form more stable biochars with highly aromatic characteristics^52^. Additionally, residual lignin in wood biochar increases its thermal stability^53^. Lignin has a higher decomposition temperature than cellulose and hemicellulose. Moreover, hemicelluloses are decomposed at 220–315 °C, and cellulose is decomposed at 315–400 °C, whereas lignin decomposition occurs at 400 °C. Thus, the average activation energy needed for breakdown may be more enormous^54^. Other research has shown similar findings^55,56^. The thermal durability of lignin ensures that the pore structure of wood biomass biochar is preserved. As a result, it has more porosity and BET Specific Surface Area^51^, consistent with the results obtained.

In all cases, the removal efficiency and Kd values of Pb(II) ions in the soil are higher than the established values for Cu(II) and Zn(II). All the sorbents were determined in 0.05–0.1 mM of metals. There were no comparable differences in the efficacy of the biochars. However, when applying high concentrations of metals up to 1 mM, the Kd values and the removal efficiency by wood biochar were higher than that by rice husks, and to a greater extent, by sunflower husks. Thus, the biochars were arranged in the following series according to their sorption capacities: wood, rice husks, and sunflower husks.

The constructed isotherms of adsorption of HM separately by soil and with the addition of biochars are shown in Fig. 6. In all cases, there is a higher affinity for Pb(II) and Cu(II) than for Zn(II), as evidenced by the shape of the isotherms. The results obtained are consistent with previous studies^57,58^. Thus, the ability of biochar from sugar beet to absorb HM decreased in the series Cd(II) > Ni(II) > Pb(II) > Cu(II), biochar obtained from dairy production waste: Pb(II) > Cu(II) > Cd(II) > Ni(II)^59^. It was reported that the ability of HM uptake by biochar obtained during the hydrothermal carbonization of peanut hulls changes in the order: Pb(II) > Cu(II) > Cd(II) > Ni(II)^16^. This is mainly due to the differences in hydrolyzability, electro-negativity, and «bond softness» according to Misono et al.^60^. McBride^61^ believed that electro-negativity is an essential factor determining the ability of a metal to chemisorption and proposed the order of placement of elements: Pb (2.33) > Cu (1.90) > Ni (1.80) > Cd (1.69) > Cr (1.66) > Zn (1.65) > Mn (1.55). Since the electro-negativity of Pb(II) is more significant than Cu(II) and Zn(II), the sorption of Pb by soil is preferable. Lead has a more excellent softness value ((Pb(3.58) > Cu (2.89) > Zn (2.34)), which determines the sequence of metal affinity or selectivity of soil components with the formation of covalent bonds^62–64^. The interaction of metal cations with the solvent (hydrolysis) also plays a role in their interaction with the adsorbent^64^. The hydrolysis constant of the first stage (pK1) is the most informative indicator of the selectivity of metal adsorption by soil. Thus, the value of pK1 varies from 7.2 to 7.8 for Pb, 7.3 to 8.0 for Cu, and 9.0–9.4 for Zn^65^. Lower values of the hydrolysis constants Pb(II) and Cu(II) compared to Zn(II) indicate their more energetic interaction with the soil due to the formation of inner-sphere complexes of metal ions^31^ or the process of sorption reactions.

The introduction of biochars obtained from different feedstocks into the soil leads to an increase in the absorption capacity of the soil concerning HM. The efficient sorption of pollutants in biochar indicates that all studied biochars (derived from wood, rice husks, or sunflower husks) sequester pollutants. The biochar made from agricultural waste outperformed the others for two reasons. Firstly, the biochar from rice husks had more micropores and total pore volume than wood's, making it more favorable for the adsorption of HM (physical adsorption). Biochar from sunflower husk had intermediate parameters. According to the literature; all the tested biochars possessed sufficient functional groups on their surface that may interact with metals through π-cation interactions^66^.

The adsorption parameters are higher for Pb(II) in all cases. The better adsorption capacity of Pb(II) ions upon introducing biochar can be explained from the Lewis-Pearson theory on the nature of the specific binding of hard and soft ions-complexing agents with the corresponding ligands, i.e., the functionally active groups of the sorbent^67^. According to the Hard and Soft, Acids and Bases (HSAB) concept hypothesis^68^, a more powerful acid Pb(II) would have a higher affinity for the hydroxyl functional groups (3100–3600 cm^−1^) and phenolic groups (900–1250 cm^−1^) than Cu(II) or Zn(II), which are soft Lewis acids. As a result, Pb(II) is a better candidate for both electrostatic and inner-sphere surface complexation processes than the other elements due to its preferred retention over Cu(II) or Zn(II).

## Conclusions

The present work addresses practical approaches using different fabricated biochars to restore HM-contaminated soils and comprehensively evaluate biochar characteristics. The origin of the feedstock influenced the composition and properties of the studied biochars. Results of elemental composition, surface measurements, sorption characteristics, and spectral and thermodynamic parameters confirm the suitability of biochars from crop waste as potential sorbents for contaminated soil remediation. The biochars' properties from various biomass sources determined their specificity in HM adsorption. The addition of biochar increased the removal efficiency of the Calcaric Fluvic Arenosols for Pb(II), Cu (II), and Zn(II). The highest adsorption ability of the biochars was observed for Pb(II) (up to 95%). Adsorption assays specified that the wood biochar had the maximum adsorption capacity for HM owing to its higher BET Specific Surface Areas and more aromatic functional groups. Biochars obtained from crop waste had comparable efficiency of sorption of HM from the soil and much greater efficiency than pure soil. The high cost of wood and the possibility of using it in many more efficient ways increase the potential for using agricultural waste. Transforming agricultural residues into biochar is a cost-effective, environmentally acceptable approach for manufacturing highly desirable carbon sorbent with a high sorption capacity that could be used to remediate contaminated soils. Using biochar resulted in the accumulation of contaminants in the amended soils. However, the environmental destiny of the sequestered pollutants over the long term is still poorly known. Studies about using biochar for the remediation of contaminated soils mainly focus on laboratory and greenhouse experiments. Largescale field trials are essential before operational scale remediation projects are implemented.

## Materials and methods

### Biochar pyrolysis method

Biochar from rice husks and sunflower husks was obtained using the stepwise pyrolysis technology proposed by the authors. This is a new method proposed by our team of authors. Pyrolysis included the following stages: drying raw materials, pyrolysis, and cooling. In this case, the pyrolysis itself is divided into three parts. The main purpose of pyrolysis is the removal of volatile substances from lignocellulosic biomass. Volatile substances (the main fraction) of lignocellulosic biomass are removed thermally (150–480° C) through the main removal reactions. Therefore, in the first part (100–300 °C), the primary removal of the volatile fraction of lignin from plant materials occurred. In the second stage (300–500 °C), the removal of volatile lignin compounds occurred more intensively. Also, at the same stage, there was a charring of raw materials. Next, the temperature was raised to 700 °C to remove lignin residues. When separating into stages, the goal was to eliminate lignin and pyrolysis products to increase the porosity and specific surface area of the resulting biochar. The final stage pyrolysis temperature of 750 °C was also considered by Hu et al.^69^. Similar pyrolysis temperatures to obtain biochar with high porosity have been described^17,53,70^. The yield of biochar from sunflower husks was 32%, and that from rice husks was 34% of the raw material weight. The biochar samples were compared with those from commercial birch wood waste purchased from Ivchar LLC, trademark A GOST 7657-84.

### Characterisation

The contents of C, H, and N (in %) of the samples were identified by high-temperature catalytic combustion using a TOC-L CPN Shimadzu analyser. Subsequently, the biochar samples were heated for 6 h at 800 °C to determine ash content, according to the following formula^71^.

1 $${\rm{Ash }}\left( {\rm{\% }} \right){ } = { }\frac{{\rm{weight \; of \; ashed \; sample}}}{{\rm{dry \; weight \; of \; sample}}} \times 100$$

The oxygen content was calculated by difference^72^.

2 $${\rm{O \% }} = { }100{ }{-}{ }\left( {{\rm{ash content }} + {\rm{ C }} + {\rm{ H }} + {\rm{ N}}} \right)$$

After determining the O content, the atomic ratios of the main elements were calculated. The elemental compositions of the powder samples of the carbonaceous sorbents were determined using X-ray fluorescence spectroscopy (XRF, Max GV, 2020, Russia).

X-ray diffraction (XRD) was utilized to establish the degree of crystallinity of the sorbents. The measurements were performed on a Bruker D2 Phaser diffractometer (20°–90° with a step of 0.01°and a scan time of 0.2 s). Five passes were performed for each sample in intensity accumulation mode, with Cu as the anode material in the LYNXEYE/SSD160 detector.

The FTIR spectra were measured on an FSM-1202 spectrometer in the transmission mode using a DTGS detector. The spectra were acquired in the 4000–400 cm^−1^. In this regard, a reference sample made of a 200 mg KBr pellet with 13 mm diameter was used. Studied samples with a mass of 0.14 mg (wood biochar), 0.21 mg (sunflower husk biochar), and 0.18 mg (rice husk biochar) were ground and milled with KBr and pressed into a pellet of a total mass of 200 mg.

The morphologies of the sorbents were studied using scanning electron microscopy (SEM, Carl Zeiss EVO-40 XVP) and confocal microscopy (KM Keyence VK 9700). The SEM survey was conducted under standard conditions for nonconductive and low-contrast samples (low vacuum, 15 kV, increased emission). The 3D modelling of the biochar sample surfaces was performed using a scanning laser microscope (Keyence VK-9700 with a violet laser wavelength of 408 nm).

The biochar samples' BET Specific Surface Area and porosity were calculated through a volumetric analyser ASAP 2020 by N_2_ adsorption–desorption. The surface and porosity factors were calculated using the Brunauer–Emmett–Teller (BET) method for N_2_ within the range of acceptable values P/P0 = 0.05–0.33.

The samples' micro -and mesopore volumes were determined by a comparative t-test using the Harkins–Jura equation. The density functional theory (NLDFT) was used to determine the pore size distribution.

The thermal stability of the materials was examined using thermogravimetric analysis (TGA) and differential scanning calorimetry (DSC) together with a thermal analyser (STA 449 F5 Jupiter, Netzsch). For analysis, the sorbent samples were placed in corundum crucibles. The measurements were performed at 25–800 °C with 70 mL/min airflow.

### Determination of the heavy metal sorption capacity of biochars

An experimental sample was taken from the surface of virgin soil (0–20 cm)—the soil type was Calcaric Fluvic Arenosol (Rostov region, Russia). The soil samples' physical and chemical properties were analyzed per the standard^73^. The following physical and chemical qualities were found in the soil: C_org_: 0.9%; pH: 7.5; exchangeable cations (Ca^2++^, Mg^2+^): 6.6 mmol( +)/100 g; cation exchangeable capacity (CEC): 6.6 mmol( +)/100 g; CaCO_3_: 0.1%; silt particles (< 0.01 mm): 2.8%; clay particles (< 0.001 mm): 1.6%; content of Cu: 15.0 mg.kg^-1^; Zn: 85.0 mg.kg^-1^; Pb: 10.9 mg.kg^-1^. Wood biochar and biochars obtained from rice husk and sunflower husk at a dose of 2.5% of the soil mass were added separately to the soil samples. Because in the pre-sorption experiments with biochar in an equilibrium solution, the concentration of metals was below the detection limit, indicating a high biochar adsorption capacity.

All studies were performed together with the soil in triplicates. The stock solutions in variable concentrations Cu (II), Zn (II), and Pb (II), were produced by dissolving standard salts of the respective metal for the adsorption study. The chemicals were stored in tightly sealed containers to avoid contact with sunlight and water. For Zn(II) metal ion solution, Zn(NO_3_)_2_·6H_2_O (Merck, Kenilworth, NJ, USA) with 98% purity was used. For Cu(II) metal ion solution, Cu (NO_3_)_2_·3H_2_O (Merck, Kenilworth, NJ, USA) with 98% purity was used. For Pb(II) metal ion solution, Pb (NO_3_)_2_ (Merck, Kenilworth, NJ, USA) with 99% purity was used in deionized water. Sorption isotherm experiments were conducted using the above solution with initial concentrations of heavy metals set as 0.05, 0.08, 0.1, 0.3, 0.5, 0.8, and 1.0 mM·L^-1^, respectively. In addition, the pH of the solutions was adjusted using a dilution of nitric acid (0.1 M) and potassium hydroxide (0.01 M) (Merck, Kenilworth, NJ, USA). The soil with biochar (5 g) was added to the heavy metal solution (50 mL), and the mixture was shaken (200 rpm, 1 h) and then left in a calm state for 24 h to reach equilibrium^74^. Studies such as^16^ prescribe contact of 24 h to reach equilibrium. Still, longer contact times have not shown significant changes in equilibrium concentration and ensure that the adsorption phase had reached equilibrium^75^. The mixture was then filtered for heavy metal analysis by Atomic Absorption Spectrophotometric technique (AAS) (The MGA-915 AA spectrometer, Lyumeks, Russia). The adsorption capacity, C_ad_ (mM∙kg^–1^), was calculated according to the following equation:

3 $${\rm{C}}_{{{\rm{ad}}}} { = }\frac{{\left( {{\rm{C}}_{{\rm{i}}} - {\rm{C}}_{{{\rm{eq}}}} } \right){\rm{ \times V}}}}{{\rm{m}}}$$

where C_i_ and C_eq_ are the initial and equilibrium solution metal concentrations, respectively (mM·L^-1^); V is the volume of the solution (ml), and m is the mass of the sorbent (kg). The information gathered was used to create the C_eq_–C_ad_ plot.

One of the main parameters of sorption capacity efficiency is the equilibrium sorption coefficient (Kd) (L·kg^−1^); Kd is commonly used in estimating the potential sorption of dissolved metals by a solid phase with higher values indicating higher sorption potential^76^.

4 $${\rm{K}}_{{\rm{d}}} { = }\frac{{\left( {{\rm{C}}_{{\rm{i}}} - {\rm{C}}_{{{\rm{eq}}}} } \right){\rm{ \times V}}}}{{{\rm{C}}_{{{\rm{eq}}}} {\rm{ \times m}}}}$$

The removal efficiency was determined by computing the percentage sorption using the following equation:

5 $$Removal \left( \% \right) = \frac{{{\rm{C}}_{{\rm{i}}} - {\rm{C}}_{{{\rm{eq}}}} }}{{{\rm{C}}_{{\rm{i}}} }}{\rm{ \times 100}}$$

The Langmuir model is based on three key hypotheses: all adsorption sites on the surface of the sorbent are energetically comparable; sorption happens in separate locations, and there is no contact between sorbed ions^40,77,78^. The Langmuir model posits that monolayer coverage occurs in a homogenous adsorption mode on a surface with binding sites with equal energies. Its maximum occurs when the monolayer is entirely saturated^79^, and it can be expressed as follows.

6 $${\rm{C}}_{{{\rm{ad}}}} = { }\frac{{{\rm{C}}_{{\rm{m}}} {\rm{K}}_{{\rm{L}}} }}{{1{ } + {\rm{ K}}_{{\rm{L}}} {\rm{C}}_{{\rm{e}}} }}$$

where C_m_ is the maximum adsorption capacity for the metal (mM∙kg^−1^); C_e_ is the concentration of metal at equilibrium (mM∙L^−1^); and K_L_ is the constant of the Langmuir model (L∙mM^−1^).

The Freundlich model suggests that sorption centers with higher adsorption capacity are filled first^61^, and the bond strength is inversely correlated with the degree of filling. In contrast to the Langmuir equation, the Freundlich isotherm does not determine the saturation limitation value for metal adsorption^80^. Multilayer and heterogeneous adsorption are taken into consideration by the Freundlich isotherm model. The following equation gives this model of an isotherm.

7 $${\rm{C}}_{{{\rm{ad}}}} {\rm{ = K}}_{{\rm{F}}} {\rm{C}}_{{\rm{e}}}^{{\rm{n}}}$$

where K_F_ is the equilibrium constant (L∙kg^–1^) and n is the function of the strength of adsorption, related to the surface heterogeneity, respectively^73^.

The Freundlich equation does not have a theoretical thermodynamic basis like the Langmuir equation. However, to some extent, it reflects the energy inhomogeneity of sorption centers. The K_F_ coefficient in the Freundlich model measures the relative absorption capacity. The dimensionless parameter n makes it possible to reveal the energy inhomogeneity of the reaction centers on the sorbing surface. It can vary in the range of 0 < n ≤ 1. When the value of n approaches 0, the inhomogeneity of the sorption centers increases to 1—decreases.

Using the Langmuir (Eq. (6)) and Freundlich (Eq. (7)) models, the adsorption data of metal ions by biochar samples were analyzed. These models are a convenient way to represent adsorption under equilibrium conditions, and the associated isotherms provide a straightforward comparison of metal affinity for various adsorbents^81^.

### Statistical analysis

All laboratory experiments were performed in triplicates. The statistical analysis was performed using SigmaPlot 12.5 and STATISTICA software packages with a confidence coefficient of 0.95. Origin was used to assist simulations of the Langmuir and Freundlich models. The correlation coefficient (R2) was used to assess the model’s accuracy.

### Ethics declarations

All methods were carried out following relevant guidelines and regulations.

## Supplementary Information

Supplementary Information.

## Data Availability

The datasets used and / or analyzed during this study are available from the corresponding author upon reasonable request.
