# Supplementary material for: Fabrication of biochar derived from different types of feedstocks as an efficient adsorbent for soil heavy metal removal
Source: Sci Rep. 2023 Feb 3;13:2020. doi: 10.1038/s41598-023-27638-9 (PMC9898244; doi:10.1038/s41598-023-27638-9)
Supplement: Supplementary file 1 — Supplementary Information. [file 41598_2023_27638_MOESM1_ESM.pdf]

Supplementary information

Supplementary Table S1. XRF analysis of different biochar samples.

Element	Rice husk	Sunflower husk	Wood
TiO_2, %	0.41	0.37	0.26
CaO, %	5.90	5.22	26.30
Al_2O_3, %	5.02	4.16	7.20
SiO_2, %	47.52	3.77	8.80
Fe_2O_3, %	3.16	3.44	4.17
P_2O_5, %	6.93	20.96	9.40
K_2O, %	0.46	24.33	3.94
MgO, %	3.33	4.20	3.47
V, mg/kg	n.d.	n.d.	n.d.
Cr, mg/kg	22.55±1.15	24.51±1.25	18.04±0.92
Mn, mg/kg	295.13±14.46	301.50±14.77	407.66±10.18
Co, mg/kg	10.64±0.47	10.97±0.48	6.52±0.29
Ni, mg/kg	12.12±0.56	15.24±0.70	20.10±0.92
Cu, mg/kg	20.51±0.87	24.23±1.02	23.47±0.99
Zn, mg/kg	34.76±1.64	29.80±1.40	27.70±1.30
Sr, mg/kg	45.70±1.89	56.49±2.33	31.07±1.28
Pb, mg/kg	n.d.	0.40±0.02	n.d.

n.d.: not detected
